# Depigmenting potential of lichen extracts evaluated by in vitro and in vivo tests

**DOI:** 10.7717/peerj.9150

**Published:** 2020-05-15

**Authors:** Paola Malaspina, Erica Catellani, Bruno Burlando, Daniele Brignole, Laura Cornara, Miriam Bazzicalupo, Simona Candiani, Valentina Obino, Vincenzo De Feo, Lucia Caputo, Paolo Giordani

**Affiliations:** 1 Department of Pharmacy, University of Genoa, Genoa, Italy; 2 Biophysics Institute, National Research Council (CNR), Genoa, Italy; 3 DISTAV, University of Genoa, Genoa, Italy; 4 DIFARMA, University of Salerno, Fisciano, Italy

**Keywords:** Tyrosinase, Lichen secondary metabolites, Zebrafish, Melanogenesis, *Letharia vulpina*, *Cetraria islandica*

## Abstract

Melanin is the main pigment of human skin, playing the primary role of protection from ultraviolet radiation. Alteration of the melanin production may lead to hyperpigmentation diseases, with both aesthetic and health consequences. Thus, suppressors of melanogenesis are considered useful tools for medical and cosmetic treatments. A great interest is focused on natural sources, aimed at finding safe and quantitatively available depigmenting substances. Lichens are thought to be possible sources of this kind of compounds, as the occurrence of many phenolic molecules suggests possible effects on phenolase enzymes involved in melanin synthesis, like tyrosinase. In this work, we used four lichen species, *Cetraria islandica* Ach., *Flavoparmelia caperata* Hale, *Letharia vulpina* (L.) Hue, and *Parmotrema perlatum* (Hudson) M. Choisy, to obtain extracts in solvents of increasing polarity, viz. chloroform, chloroform-methanol, methanol, and water. Cell-free, tyrosinase inhibition experiments showed highest inhibition for *L. vulpina* methanol extract, followed by *C. islandica* chloroform-methanol one. Comparable results for depigmenting activities were observed by means of in vitro and in vivo systems, such as MeWo melanoma cells and zebrafish larvae. Our study provides first evidence of depigmenting effects of lichen extracts, from tyrosinase inhibition to cell and in vivo models, suggesting that *L. vulpina* and *C. islandica* extracts deserve to be further studied for developing skin-whitening products.

## Introduction

In vertebrates, melanin synthesis is realized by specialized cells called melanocytes, within lysosome-like organelles called melanosomes. Melanization is controlled by different processes, including environmental (e.g., UV rays) and endogenous (e.g.,  α-MSH) factors, stimulation of melanocortin-1 receptor (MC1R), signal transduction by cAMP and MAPK pathways, activation of microphthalmia-associated transcription factor (MITF), and expression of premelanosome protein (Pmel), tyrosinase (TYR), and tyrosinase-related proteins (TYRP1) (D’Mello et al., 2016; Cheli et al., 2010).

Tyrosinase (EC1.14.18.1) is a key enzyme of melanin synthesis and has been widely investigated as a target of modulatory agents of melanization. It is a multifunctional copper-containing enzyme, widely distributed in nature, responsible for melanization in animals and for browning in plants and microorganism (Kondo & Hearing, 2011). The enzyme catalyzes two distinct reactions of melanin formation: hydroxylation of tyrosine by monophenolase activity, and oxidation of 3,4-dihydroxyphenylalanine (L-DOPA) to *o*-dopaquinone by diphenolase action. These reactive *o*-quinones undergo non-enzymatical polymerization to form melanin.

Although melanin in human skin is an essential pigment for the protection against UV-induced damage, excessive melanin production causes hyperpigmentation disorders, such as melasma, ephelides, and lentigines (Mukherjee et al., 2018). These conditions represent a problem for many people and as a consequence the search for depigmenting agents has attracted much interest in the medical and pharmaceutical fields (Solano, 2014). Therefore, considerable research effort has been directed to discover new natural active products rich in safe and quantitatively available inhibitors of pigmentation (Li et al., 2013; Lo et al., 2013; Wang et al., 2011). A main strategy is to target tyrosinase, with an increasing interest for natural products to be used as tyrosinase inhibitors (Leyden et al., 2011). In literature, a large number of tyrosinase inhibitors from natural sources have been reported for their possible use to treat pigmentation skin disorders (Mukherjee et al., 2018; Parvez et al., 2007). For many of these compounds the inhibitory activity has been related to their phenolic structure that provides high antioxidant power. Various pieces of evidence indicate that lichens are worth being investigated as possible sources of this kind of compounds (Brandão et al., 2017; Higuchi et al., 1993; Honda et al., 2016; Lopes, Coelho & Honda, 2018).

Lichens are symbiotic associations between a heterotrophic fungus (the mycobiont) and one or more photosynthetic partners (the photobionts) (Nash III, 2006). As a result of the symbiosis, the mycobiont produces several secondary metabolites (called lichen substances), most of which are unique to these organisms (Ranković & Kosanić, 2015). These metabolites may help to protect against biotic and abiotic factors, such as herbivores or UV radiation (Phinney, Solhaug & Gauslaa, 2018). Most of them are phenolic compounds derived mainly from the acetate-polymalonate pathway and are expected to afford several biological activities. Accordingly, lichens are used in traditional medicines across the world for different purposes (Crawford, 2015; Devkota et al., 2017), while different lichen substances have been taken up for herbal and pharmaceutical applications (Einarsdóttir et al., 2010; Gülçin et al., 2002; Ranković & Kosanić, 2015). Among different properties, the phenolic nature of these compounds suggests effects on the activity of phenolase enzymes like tyrosinase (Honda et al., 2016). Lichens are good sources of natural antioxidants, some of which recognized as tyrosinase inhibitors (Behera, Adawadkar & Makhija, 2004). Some patents are also claiming activities against tyrosinase related to lichen extracts or lichen compounds (Takayama et al., 2010), but they have been long neglected and overlooked principally due to difficulties in obtaining lichen substances in quantities and purities sufficient for structural elucidation and pharmacological testing (Boustie, Tomasi & Grube, 2011; Muggia, Schmitt & Grube, 2009).

This study was aimed at exploring the biological effects of lichen substances on various pigmentation models, both cell-free and cellular ones, also including in vivo Zebrafish experiments. Moreover, we provided hints about the possibility of exploiting lichens for the extraction of depigmenting compounds and the preparation of pharmaceutical and cosmetic depigmenting products. Due to limited knowledge of lichen effects on melanization, we firstly performed a screening conducted on different species known for their wide distribution and abundance, viz. *Cetraria islandica* Ach., *Flavoparmelia caperata* Hale, *Letharia vulpina* (L.) Hue, and *Parmotrema perlatum* (Hudson) M. Choisy. From each species, we obtained a series of four extracts by using solvents of increasing polarity, from pure chloroform to water. As a first survey, all extracts were tested in cell-free, tyrosinase inhibition experiments. The extracts showing marked dose-dependent inhibitory activity were used on in vitro and in vivo melanization models, by using cultures of melanin-producing melanoma cells (MeWo), and developing zebrafish embryos, respectively. We used zebrafish because it has been recently established as an in vivo model for phenotype-based screening of melanogenic regulatory compounds (Lin et al., 2011). In particular, zebrafish has become an important vertebrate model for assessing drug effects because it exhibits unique features, including ease of maintenance and drug administration, short reproductive cycle, external fertilization and development, allowing manipulation of the developmental environment and optical measurements through the transparent body wall.

## Materials and Methods

### Chemicals

All reagents were purchased from Sigma-Aldrich (Milan, Italy), unless otherwise indicated.

### Lichen species and extract preparation

Thalli of *F. caperata* and of *P. perlatum* were collected in a woodland area of eastern Liguria (NW Italy), *L. vulpina* in a forest area of Valtournenche (NE Valle d’Aosta, Italy), and *C. islandica* was purchased from Kubja Ürditalu (Tallinn, Estonia). No permissions for lichen collection are required according to Italian legislation. Lichen materials were identified by one of us (PG) using microscope analysis with the help of identification keys and spot tests. Thereafter lichen material was cleaned from debris, left to dry at room temperature overnight, and stored in paper bags at room temperature until use.

Dried lichen thalli were extracted at room temperature (about 23 °C) with four solvent polarities from chloroform to water: chloroform, chloroform–methanol (9:1), methanol, and water (14.4 g of *F. caperata* in 70 mL of each solvent, 10.4 g of *P. perlatum* in 60 mL, 13.3 g of *L. vulpina* in 75 mL, and 100.6 g of *C. islandica* in 500 mL). Extractions were carried out for 5 days and 3 times for each solvent, with frequent agitation. The supernatant liquid was then filtered and evaporated to dryness under reduced pressure in a rotary system (Rotavapor Heidolph, Schwabach, Germany) to obtain dried extracts (Souza et al., 2016). Lichen extract yields are reported in Table 1.

**Table 1:** Lichen extract yields (%) obtained by using different solvents.

Lichen species	CHCl_3_	CHCl_3_/CH_3_OH		CH_3_OH	H_2_O
*Flavoparmelia caperata*	2.3		3.0	6.8	0.6
*Parmotrema perlatum*	1.6		5.6	5.3	0.5
*Cetraria islandica*	1.3		1.5	3.7	0.5
*Letharia vulpina*	1.2		1.9	4.4	4.0

### Tyrosinase inhibition assay

Lichen extracts were dissolved in dimethyl sulfoxide (DMSO) to a final concentration of 10 mg/ml. Extract stock solutions were then diluted in water in order to obtain a series of test solutions with final concentrations of 10, 50, 100, 250, 350 and 500 µg/ml. Components of the reaction mix were added to each well of 96-well plates in the following order: 70 µL of phosphate buffer, 60 µL of extract solutions (water for controls), 10 µL of mushroom tyrosinase (Sigma-Aldrich, T3824, 25 kU, 125 U/ml in phosphate buffer, pH 6.8) and 70 µL L-tyrosine (0.3 mg/ml in water). Kojic acid was used instead of lichen extracts as a positive control, at increasing concentrations from 0.5 to 500 µg/ml. Blank samples without enzyme were also included for all conditions. Plates were then incubated at 30 °C for 60 min and absorbance was read at 505 nm in a microplate reader (Spectra Max 340PC). Percent inhibitory activity (I%) was calculated according to the formula

\begin{eqnarray*}\mathrm{I}\text{%}= \left[ 1- \frac{ \left( {\mathrm{A}}_{\mathrm{ex}/\mathrm{en}}-{\mathrm{A}}_{\mathrm{ex}} \right) }{ \left( {\mathrm{A}}_{\mathrm{en}}-{\mathrm{A}}_{\mathrm{bk}} \right) } \right] \times 100 \end{eqnarray*}

where A_ex∕en_ = absorbance of sample mixture with extract and enzyme; A_ex_ = absorbance of sample mixture with extract and without enzyme; A_en_ = absorbance of sample mixture with enzyme and without extract; A_bk_ = absorbance of sample mixture without enzyme and extract (blank).

### TLC bioautography analysis

Conventional TLC chromatographic profile was conducted on 20 × 20 cm plates (Merck silica gel 60 F_254_) following literature protocols (Culberson & Kristinsson, 1970; White & James, 1985). Briefly, samples of *L. vulpina* methanolic extract and of *C. islandica* chloroform-methanol extract were resolubilized in their extraction solvents and then spotted on a TLC plate using a capillary tube. TLC profile was carried out using toluene:acetic acid (200:30 ml) as a mobile phase (solvent C). After the solvent front was reached, the plate was left to dry at room temperature. Dried plates were examined and photographed initially in visible light (daylight) for detecting pigments as colored spots, and then in fluorescence light, using 254 and 350 nm excitations.

To visualize the presence of tyrosinase inhibition activity in each spot, TLC plates were sprayed with L-tyrosine solution (about 2.5 × 10^−5^ mmol/cm^2^) and then with tyrosinase solution (about 3.6 U/cm^2^). Spots with tyrosinase inhibitory activity appeared white on a dark background (Wangthong et al., 2007).

### Cell culture, cell viability, and melanin assays

The MeWo human melanoma cell line (cat. HTB-65, ATCC, Manassas, VA, USA, https://www.lgcstandards-atcc.org/products/all/HTB-65.aspx) was used for cell viability assay, as reported by Pastorino et al. (2017), and for melanin assay, as described by Cornara et al. (2018).

Briefly, for cell viability assay, cells were settled in 96-well plates, exposed for 48 h to a logarithmic series of lichen extract concentrations, probed with 3-(4,5-dimethylthiazol-2-yl)-2,5-diphenyltetrazolium bromide (MTT) reaction, and read at 550 nm in a microplate reader (Spectra Max 340 PC). For melanin assay, cells were settled in 24-well plates, exposed in triplicate to lichen extracts, or arbutin (8 mM) as positive control, washed with PBS, trypsinized, centrifuged, freeze-thawed, dissolved in 1 N NaOH and read at 505 nm in the microplate reader. In particular, non-cytotoxic concentrations were tested: 10 and 50 µg/ml for *L. vulpina*, and 25 and 50 µg/ml for *C. islandica*.

### Zebrafish depigmentation assay

Adult zebrafish (*Danio rerio*) were obtained from a commercial dealer and kept in a circulating system with water conductivity of 500–530 Ω/cm at 27 °C, pH 7.0–7.5 under a constant 14/10 light/dark photoperiod. Veterinary care of the animals was carried out according to the Italian law (D.to L.Vo 26/2014) and the experiments were approved by the Institutional Ethics Review Body (University of Genoa) and the Italian ministry of Health (authorization 720/2015-PR). Spawning of adult zebrafish was performed according to standard methods. In particular, synchronized embryos were obtained from natural spawning induced in the morning by turning on light. Embryos were arrayed in 6-well plates containing 2 ml of embryo medium and 15 embryos per well. Extract stock solutions were diluted to the desired concentrations with embryo medium just before use (*L. vulpina* methanol extract: 6–45 µg/ml; *C. islandica* chloroform-methanol extract: 5–65 µg/ml). Diluted extracts were added to each well and incubated from 8 to 56 hpf (hours post-fertilization), resulting in 48 h exposure. Arbutin 10 mM was used as positive control. Replacement of the medium was done every 24 h to ensure even distribution of the test compounds. Embryos at 56 hpf were dechorionated by forceps, anesthetized in tricaine methanesulfonate solution (Sigma-Aldrich) and then photographed under a stereomicroscope (Leica M205C). The effects on pigmentation were evaluated using the ImageJ software v. 1.74. The pixel measurement analyzer function was used to evaluate the area of zebrafish pigmentation.

### Statistical analysis

Dose–response curves obtained by tyrosinase inhibition, MTT, and zebrafish pigmentation data, were analyzed by a logistic regression model, yielding IC_50_ and IC_05_ values that were assumed as median and threshold levels, respectively. Statistic comparisons were done with R 3.0.1 (R Core Team, 2013) environment, using ANOVA test, t Student with Bonferroni’s correction and Dunnett’s tests for multiple comparisons.

## Results

### Effects on tyrosinase activity

**Figure 1:**
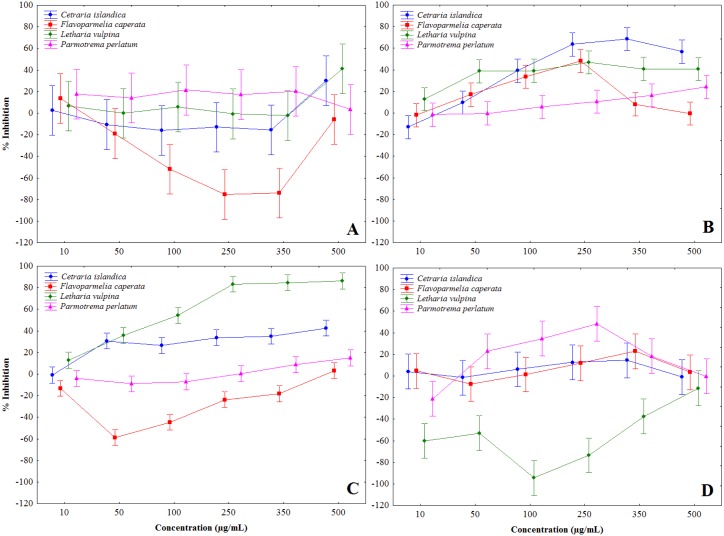
Percent inhibition of mushroom tyrosinase activity induced by lichen extracts. Percent inhibition of mushroom tyrosinase activity, induced by chloroform (A), chloroform-methanol (B), methanol (C) and water (D) extracts, obtained from *Letharia vulpina*, *Cetraria islandica*, *Parmotrema perlatum*, and *Flavoparmelia caperata*.

Lichen extracts showed a complex of modulatory effects on mushroom tyrosinase activity, evaluated in vitro in a cell-free assay. Some extracts induced tyrosinase activation, notably *F. caperata* methanol and chloroform, and *L. vulpina* water extracts, others showed a biphasic behavior, while some were definitely inhibitory (Fig. 1 and Table 2). In particular, chloroform-methanol (Fig. 1B) and methanol extracts (Fig. 1C) showed on the whole stronger tyrosinase inhibition, with dose-dependent effects. The strongest inhibition activity was recorded for the methanol extract of *L. vulpina* (Fig. 1C), followed by the chloroform-methanol extract of *C. islandica* (Fig. 1B). The inhibitory activities of these extracts were the only ones that allowed to estimate IC_50_ values with 95% CI (Table 2). As a positive control, in these experiments kojic acid induced tyrosinase inhibition with an IC50 of 13.9 µg/ml (95% confidence interval: 12.4–15.7).

**Table 2:** Values of IC_50_ (µg/ml) obtained for lichen extract inhibition on tyrosinase activity. Values calculated with more precision are accompanied by 95% CI (in parentheses).

Lichen species	CHCl_3_	CH_3_OH /CHCl_3_	CH_3_OH	H_2_O
*Letharia vulpina*	>500	>500	67 (52–86)	n.d.
*Cetraria islandica*	>500	86 (61–103)	>500	>500
*Parmotrema perlatum*	n.d.	>500	>500	biphasic^a^
*Flavoparmelia caperata*	n.d.	biphasic	>500	>500

**Notes.**

a Non-monotone trend of the dose-response curve (see also Fig. 1).

Based on the results of cell-free experiments, we selected the *L. vulpina* methanol and *C. islandica* chloroform-methanol extracts for testing their depigmenting effects on melanoma cells as an in vitro model, as well as on zebrafish as an in vivo model.

### Detection of tyrosinase inhibition by TLC bioautography

**Figure 2:**
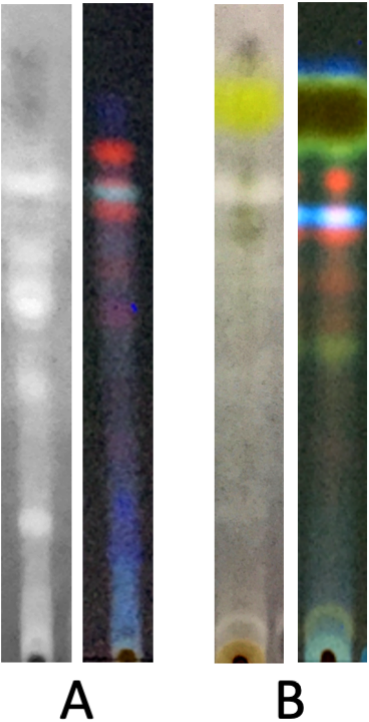
Bioautography assay of the lichen extracts along with TLC profile visualized at 350 nm. Representative lanes of TLC plates subjected to bioautography assay of the chloroform-methanol extract of *C. islandica* (A) and of the methanol extract of *L. vulpina* (B). For each lichen species, TLC plates have been observed under fluorescence at 350 nm (right lane), and under visible light after bioautography (left lane), showing white spots corresponding to compounds with tyrosinase inhibition (see Materials and Methods for technical details).

The TLC profile made it possible to show the main substances contained in each extract. An identification of these substances is not an aim of this work, but, as an example, the spot of vulpinic acid (yellow under visible light) in *L. vulpina* methanolic extract is clearly evident (Huneck & Yoshimura, 1996) (Fig. 2). Bioautography data revealed that several compounds exert inhibitory effects on tyrosinase activity. Most notably, the inhibitory activity of *C. islandica* chloroform-methanol extract was distributed among different bands covering a wide range of polarity (Fig. 2A). Conversely, the tyrosinase inhibitory activity of *L. vulpina* methanol extract is concentrated in one band that migrates close to the large yellow band corresponding to vulpinic acid (Fig. 2B).

### Lichen extract depigmenting effects on MeWO cells

The MeWo human melanoma cell line was used as an in vitro model to explore lichen depigmenting effects. As a first step, cells were subjected to cell viability assay upon exposure for 48 h to increasing concentrations of *L. vulpina* methanol and *C. islandica* chloroform-methanol extracts. Dose–response curves of cell viability allowed deriving IC_50_ values of 88 µg/ml (95% CI [68–113 µg/ml]) for *L. vulpina*, and of 264 µg/ml (95% CI [213–328 µg/ml]) for *C. islandica*. Threshold IC_05_ values were 19 µg/ml (95% CI [9–40 µg/ml]) and 51 µg/ml (95% CI [31–85 µg/ml]), respectively.

**Figure 3:**
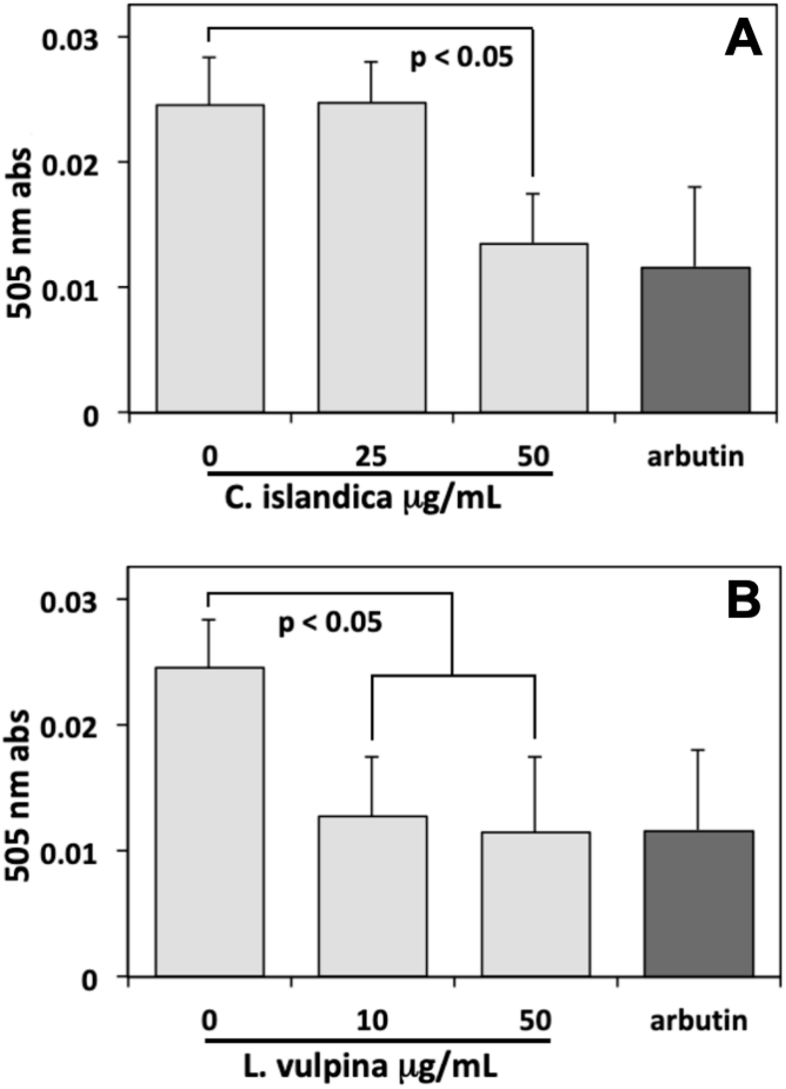
Melanin assay performed on MeWo melanoma cells exposed to lichen extracts. Melanin assay performed on MeWo melanoma cells exposed for 72 h to different concentrations of *C. islandica* chloroform-methanol (above), or *L. vulpina* methanol (below) extracts. Arbutin (8 mM) was used as positive control. Data are means ± s.d. of 505 nm absorbances derived from three independent samples read in duplicate.

Thereafter, the assay of melanin conducted on MeWo cells, after 72 h exposure to different lichen extract concentrations, showed a sharp reduction with respect to controls induced by both extracts. In these experiments, arbutin (8 mM) was used as a positive control, reducing the melanin content of cells to about 50% of controls. The methanol extract of *L. vulpina* induced a melanin reduction similar to that of arbutin, occurring already at a concentration as low as 10 µg/ml (Fig. 3). This concentration is lower than the threshold for cytotoxic effects measured for this extract, allowing to rule out the possibility of aspecific injurious effects on cells. A similar effect on the melanin content of cells was also observed for the *C. islandica* chloroform-methanol extract, but only at a concentration of 50 µg/ml (Fig. 3). However, also the effective concentration of this extract was lower than the threshold for cytotoxic effects.

### Phenotype-based evaluation of depigmenting effects of lichen extracts using zebrafish

Zebrafish models were used to further substantiate in vivo the effects of the inhibition of melanogenesis by *C. islandica* and *L. vulpina*. In order to define the optimal concentration to use, first embryos were subjected to toxicity assay upon exposure for 48 h to increasing concentrations of *L. vulpina* methanol and *C. islandica* chloroform-methanol extracts.

**Figure 4:**
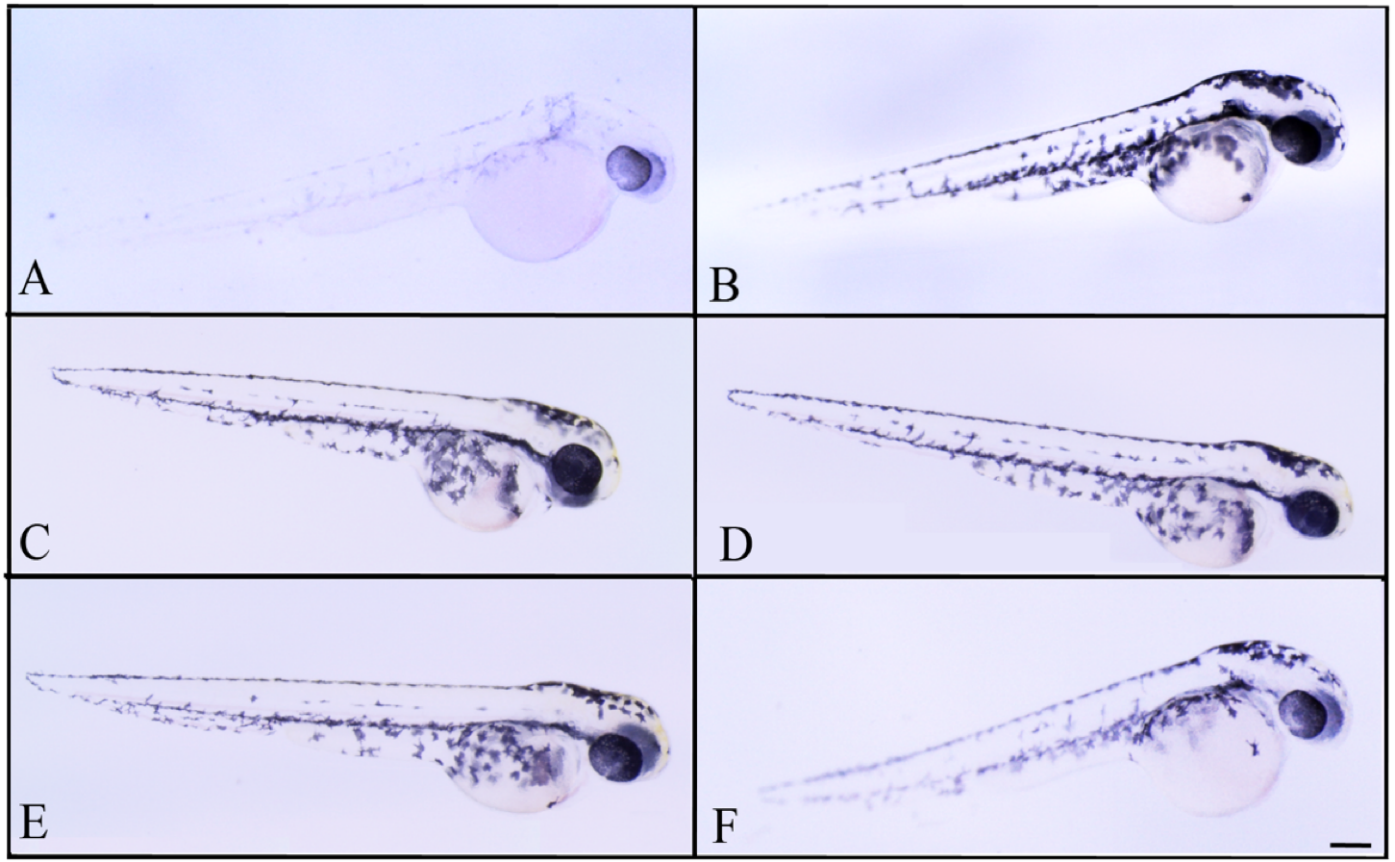
Effects of melanogenic inhibition on the pigmentation of zebrafish treated with *C. islandica* extracts. Effects of melanogenic inhibition on the pigmentation of zebrafish treated from 8 hpf to 56 hpf with *C. islandica* extract. All images are oriented to have rostral to the right and dorsal at the top. Bright field images of embryos treated with (A) arbutin (10 mM) used as a positive control; untreated zebrafish used as negative control (B); *C. islandica* chloroform-methanol extract: 5 µg/ml (C); 25 µg/ml (D); 50 µg/ml (E); 65 µg/ml (F). Scale bar: 100 µm.

**Figure 5:**
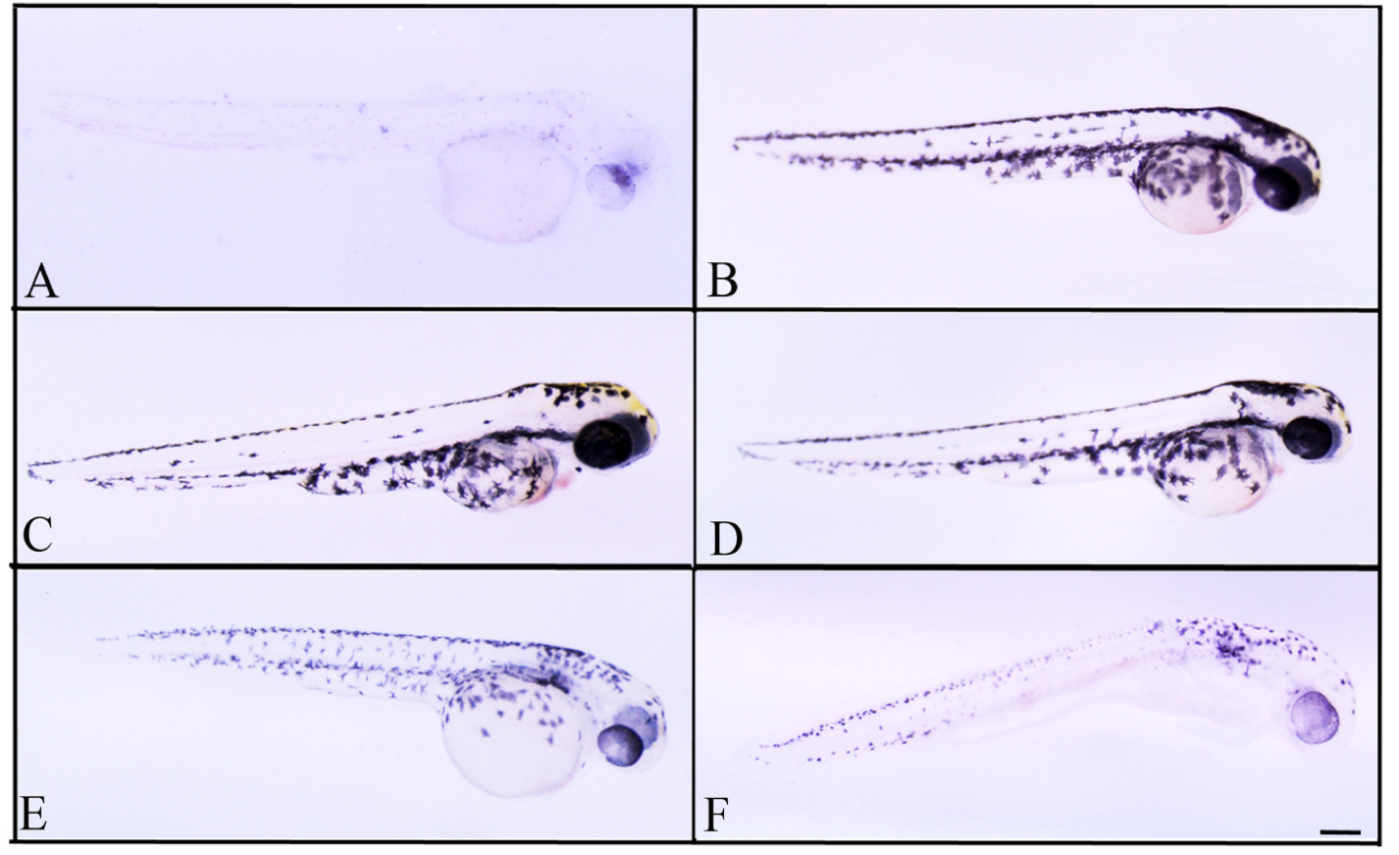
Effects of melanogenic inhibition on the pigmentation of zebrafish treated with *L. vulpina* extracts. Effects of melanogenic inhibition on the pigmentation of zebrafish treated from 8 hpf to 56 hpf with *L. vulpina* extract. All images are oriented to have rostral to the right and dorsal at the top. Bright field images of embryos treated with (A) arbutin (10 mM) used as a positive control; untreated zebrafish used as negative control (B); *L. vulpina* methanol extract: 6 µg/ml (C); 12 µg/ml (D), 22.5 µg/ml (E), 45 µg/ml (F). Scale bar: 100 µm.

**Figure 6:**
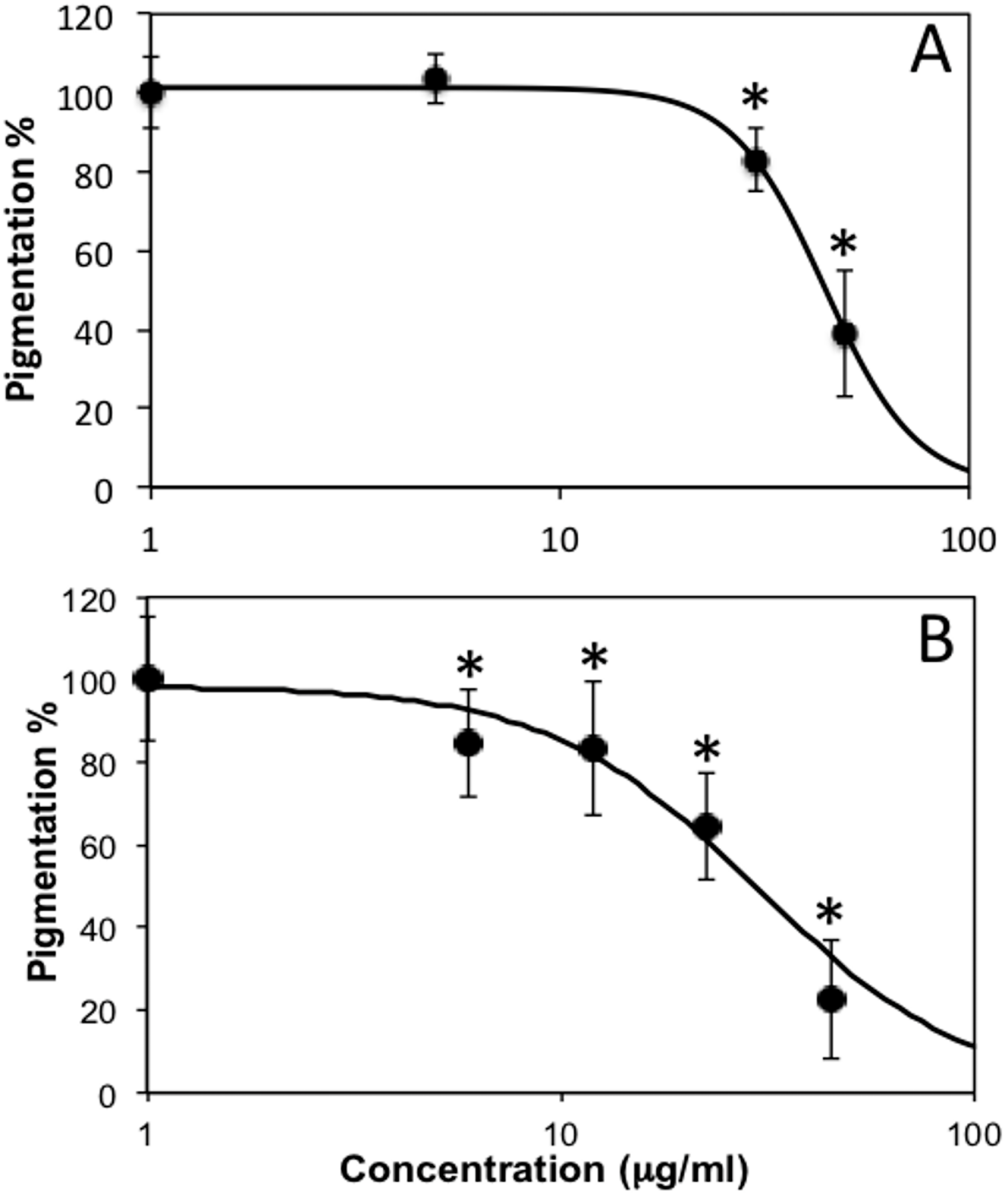
Dose response curves obtained with image analysis data of zebrafish pigmentation after exposure to different lichen extracts. Dose response curves obtained with image analysis data of zebrafish pigmentation after exposure to different lichen extracts (see Figs. 4–5): (A) *C. islandica* chloroform-methanol extract, (B) *L. vulpina* methanol extract. Dots indicate means ± s.d., *n* = 12 − 60; continuous lines indicate logistic regression curves for determination of IC_50_ values. * =*p* < 0.01 according to Dunnett’s test.

Thereafter, we observed that, when treated with subtoxic doses of *C. islandica* and *L. vulpina*, zebrafish larvae had a reduction in the pigmentation (Figs. 4 and 5). The extract of *L. vulpina* showed higher inhibitory activity than *C. islandica*, as indicated by data from image analysis. Logistic regression curves yielded IC_50_ values of 44 µg/ml (42–47 µg/ml) for the chloroform-methanol extract of *C. islandica*, and of 30 µg/ml (25–36 µg/ml) for the methanol extract of *L. vulpina* (Fig. 6). Finally, depigmenting activity of *C. islandica* and *L. vulpina* extracts was also evaluated in the zebrafish embryos by melanin assay (Supplemental Information).

## Discussion

Our study highlighted a complex of in vitro and in vivo depigmenting effects due to specific lichens and extraction solvents. The strategy to choose separate extractions with a variety of solvent polarities, instead of a successive extraction with solvents of increasing polarities, was dictated by the pioneering aspect of the investigation. The study was aimed at disclosing widely available lichen species that could be exploited for their depigmenting effects, with a very limited knowledge about the possible presence of active principles and their interactions. Therefore, we adopted an extract fractionation method that can involve some composition overlap among fractions, but maximizes their depigmenting performance, possibly also due to synergistic effects.

As for tyrosinase inhibition in cell-free experiments, our results confirm data from Higuchi et al. (1993), showing tyrosinase inhibition rates of 40.4% for *L. vulpina* and of 13.8% for *C. islandica*, with respect to our 86.2% and 42.6%, respectively, possibly due to the use of cultured lichens and different extraction solvent. We showed strongest activity for the methanol extract of *L. vulpina*, followed by the chloroform-methanol extracts of *C. islandica*. Thus, these extracts were used to explore the antimelanogenic activity on melanoma cells and on zebrafish larvae. Data obtained from these tests confirmed those from cell-free experiments, and in all cases the methanol extract of *L. vulpina* induced the strongest effect.

In addition, bioautography assay indicate that different substances contained in these lichens exert tyrosinase inhibition. Although we did not perform a complete characterization of the extracts, we know from literature the main lichen substances characterizing these lichens: *L. vulpina* contains atranorin and vulpinic acid, while *C. islandica* contains lichesterinic, protolichesterinic and fumarprotocetraric acid (Culberson, 1969). However, information regarding the antityrosinase activities of lichen substances in the literature is relatively poor, while only in a few cases it was possible to clarify the mechanisms of inhibition. Recently, Brandão et al. (2017) isolated fumarprotocetraric acid from the lichen *Cladonia verticillaris* and showed uncompetitive, mixed-type inhibition on tyrosinase activity which rose with increasing concentration, at 0.6 mM the acid inhibited tyrosinase activity by 39.8%.

An element that makes comparisons among different lichen species a difficult task is the high variability of chemical composition, which is also subjected to the variation of environmental parameters, habitat and microclimatic characteristics (e.g., availability of water and light) (Matteucci et al., 2017). These differences may underlie considerable differences in the biological activity of lichen phytocomplexes in which the composition has not been quantitatively characterized. Therefore, further work is necessary to isolate and quantify active compounds from the extracts in order to better define the components with antityrosinase activity. So far, several works have investigated the possible antityrosinase activity of lichen compounds (e.g., Kwong et al., 2020; Honda et al., 2016; Lopes, Coelho & Honda, 2018). For example, Kim & Cho (2007) determined that methanolic extracts of *Usnea longissima* and *Usnea esculenta* affected melanin formation independently from their antioxidant action. In relation to their phenolic structure, different constituents are likely to be strong tyrosinase inhibitors, with a much lower IC_50_ with respect to that of the whole extract.

In conclusion, our study provides evidence of depigmenting effects of specific lichen extracts, going from tyrosinase inhibition in cell-free experiments to depigmenting effects in vitro on cultured cells and in vivo on zebrafish larvae. These data indicate that *L. vulpina* and *C. islandica* lichen extracts are potential candidates for developing pharmaceutical and cosmetic products for skin whitening. Moreover, data also suggest that *L. vulpina* could be a good source for the isolation of compounds with strong depigmenting properties. Future objectives in this direction will be the chemical characterization of the lichen extracts and the evaluation of the activity of their most promising constituents.

##  Supplemental Information

10.7717/peerj.9150/supp-1 Supplemental Information 1 Dose-response variations of cell viability in MeWo cells Dose-response variations of cell viability in MeWo cells exposed for 48 h to increasing concentrations of *Cetraria islandica* chloroform-methanol, and to *Letharia vulpina* methanol extracts, and then subjected to MTT assay. Data are means ± s.d. of 550 nm reading. Dose-response curves allowed deriving IC50 values of 88 µg/ml (95% C.I. [68–113] µg/ml) for *L. vulpina*, and of 264 µg/ml (95% C.I. [213–328] µg/ml) for *C. islandica*.

10.7717/peerj.9150/supp-2 Supplemental Information 2 Percentage of inhibition of tyrosinase activities performed by different lichen extracts

## Additional Information and Declarations

### Data Availability

The following information was supplied regarding data availability: The raw measurements of tyrosinase inhibition are available in a Supplemental File.
