# Supplementary material for: Depigmenting potential of lichen extracts evaluated by in vitro and in vivo tests
Source: PeerJ. 2020 May 15;8:e9150. doi: 10.7717/peerj.9150 (PMC7233272; doi:10.7717/peerj.9150)
Supplement: Supplemental Information 1 — Dose-response variations of cell viability in MeWo cells exposed for 48 h to increasing concentrations of Cetraria islandica chloroform-methanol, and to Letharia vulpina methanol extracts, and then subjected to MTT assay. Data are means ± s.d. of 550 nm reading. Dose-response curves allowed deriving IC50 values of 88 µg/ml (95% C.I. [68–113] µg/ml) for L. vulpina, and of 264 µg/ml (95% C.I. [213–328] µg/ml) for C. islandica. [file peerj-08-9150-s001.pdf]

Supplementary data

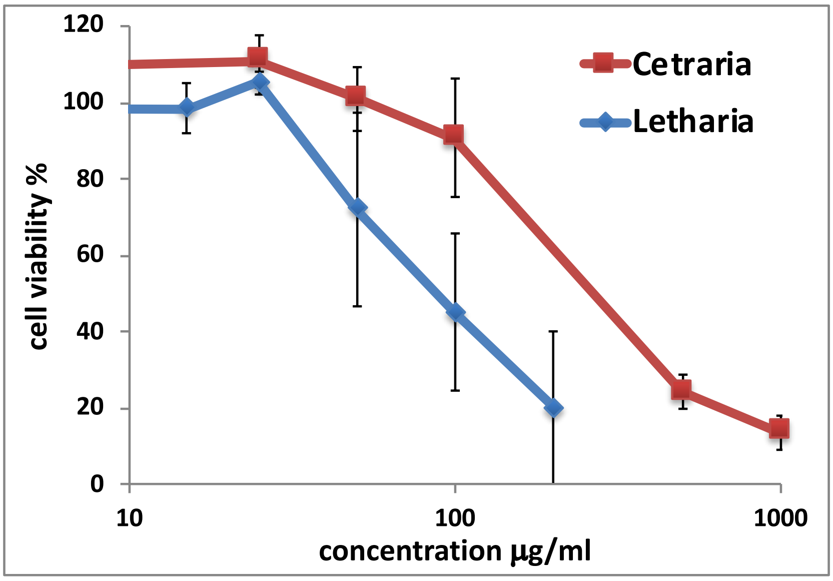

Dose-response variations of cell viability in MeWo cells exposed for 48 h to increasing concentrations of *Cetraria islandica* chloroform-methanol, and to *Letharia vulpina* methanol extracts, and then subjected to MTT assay. Data are means ± s.d. of 550 nm reading. Dose-response curves allowed deriving IC50 values of 88 µg/ml (95% C.I. = 68-113 µg/ml) for *L. vulpina*, and of 264 µg/ml (95% C.I. = 213-328 µg/ml) for *C. islandica*.

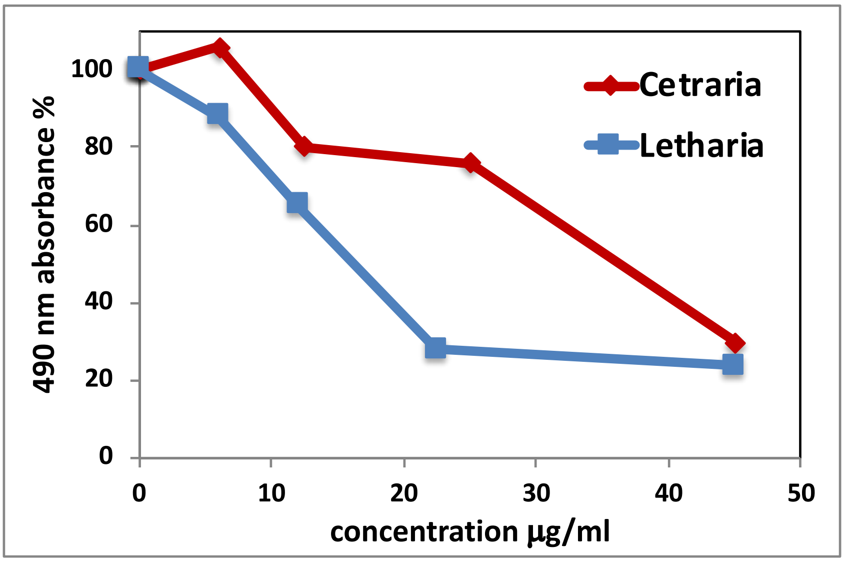

Depigmenting activity of *C. islandica* chloroform-methanol, and *L. vulpina* methanol extracts in the zebrafish embryos was also evaluated by melanin assay following Choi et al. 2007 (Choi TY, Kim JH, Ko DH, Kim CH, Hwang JS, Ahn S, Kim SY, Kim CD, Lee JH, Yoon TJ. Zebrafish as a new model for phenotype-based screening of melanogenic regulatory compounds. Pigment Cell Res. 2007 Apr;20(2):120-7). Groups of 50 zebrafish embryos exposed to extracts as well as untreated embryos were sonicated in Pro-Prep protein extraction solution. Each lysate was clarified by centrifugation at 10.000 g for 10 min and then dissolved in 1ml of 1N NaOH at 100°C for 30 min. The sample was then vortexed to solubilize the melanin pigment and the absorbance at 490 nm was measured.
